# Birth Weight and Adult IQ, but Not Anxious-Depressive Psychopathology, Are Associated with Cortical Surface Area: A Study in Twins

**DOI:** 10.1371/journal.pone.0129616

**Published:** 2015-06-18

**Authors:** Aldo Córdova-Palomera, Mar Fatjó-Vilas, Carles Falcón, Nuria Bargalló, Silvia Alemany, Benedicto Crespo-Facorro, Igor Nenadic, Lourdes Fañanás

**Affiliations:** 1 Unidad de Antropología, Departamento de Biología Animal, Facultad de Biología and Instituto de Biomedicina (IBUB), Universitat de Barcelona, Av. Diagonal, 643, 08028, Barcelona, Spain; 2 Centro de Investigaciones Biomédicas en Red de Salud Mental (CIBERSAM), C/Doctor Esquerdo, 46, 28007, Madrid, Spain; 3 Medical Image Core Facility, the Institut d’Investigacions Biomèdiques August Pi i Sunyer (IDIBAPS); C/Rosselló, 149–153, 08036, Barcelona, Spain; 4 Centro de Investigación Biomédica en Red en Bioingeniería, Biomedicina y Nanomedicina (CIBER-BBN), C/ Poeta Mariano Esquillor, s/n., 50018, Zaragoza, Spain; 5 Centro de Diagnóstico por Imagen, Hospital Clínico, C/Villarroel, 170, 08036, Barcelona, Spain; 6 University Hospital Marqués de Valdecilla, Department of Psychiatry, School of Medicine, University of Cantabria, Av. Valdecilla, s/n, 39008, Santander, Cantabria, Spain; 7 IFIMAV, Instituto de Formación e Investigación Marqués de Valdecilla, Av. Valdecilla, s/n, 39008, Santander, Cantabria, Spain; 8 Department of Psychiatry and Psychotherapy, Jena University Hospital, Friedrich Schiller University Jena, Philosophenweg 3, 07743, Jena, Germany; Radboud University, NETHERLANDS

## Abstract

**Background:**

Previous research suggests that low birth weight (BW) induces reduced brain cortical surface area (SA) which would persist until at least early adulthood. Moreover, low BW has been linked to psychiatric disorders such as depression and psychological distress, and to altered neurocognitive profiles.

**Aims:**

We present novel findings obtained by analysing high-resolution structural MRI scans of 48 twins; specifically, we aimed: i) to test the BW-SA association in a middle-aged adult sample; and ii) to assess whether either depression/anxiety disorders or intellectual quotient (IQ) influence the BW-SA link, using a monozygotic (MZ) twin design to separate environmental and genetic effects.

**Results:**

Both lower BW and decreased IQ were associated with smaller total and regional cortical SA in adulthood. Within a twin pair, lower BW was related to smaller total cortical and regional SA. In contrast, MZ twin differences in SA were not related to differences in either IQ or depression/anxiety disorders.

**Conclusion:**

The present study supports findings indicating that i) BW has a long-lasting effect on cortical SA, where some familial and environmental influences alter both foetal growth and brain morphology; ii) uniquely environmental factors affecting BW also alter SA; iii) higher IQ correlates with larger SA; and iv) these effects are not modified by internalizing psychopathology.

## Introduction

Human neurodevelopment is a highly intricate, stage-dependent, dynamic, lifetime process. Early periods of growth are of particular importance due to their enduring impact on the remaining sequence of anatomical maturational processes. In fact, intrauterine and neonatal brain insults have been shown to have a long-term impact on behaviour and neurological outcomes [1,2].

Factors such as prematurity and very low birth weight (BW) have been related to altered cortical brain features later in life [3–5]. As genetic and environmental influences modify brain features differently across stages [6] Bystron et al., 2008), studying which early modifications of the human cortex have long-lasting effects–and their potential origins, both genetic and non-genetic–may shed light on human brain maturational processes and their consequences for cognitive functioning, and mental health and disease.

Accordingly, models of developmental vulnerability to adult psychopathology have recently been fostered by twin and magnetic resonance imaging (MRI) studies. They have demonstrated that both genetic and environmental influences play a role in abnormal neurocognition and related mental health issues [7]. Genetically informative neuroimaging approaches have contributed considerably to the discipline of developmental psychopathology, since they allow links between genes, brain structure/function, and neurocognitive profiles associated with both normal and pathological psychological traits to be ascertained [8–10].

More explicitly, three recent MRI studies have consistently shown that BW variation within a normal range has an effect on cortical features detectable in children, adolescents and young adults. This effect seems to be environmentally driven and independent of major psychiatric diagnoses. Specifically, the findings indicate that BW has long-term consequences on total and regional cortical surface area (SA), but not on cortical thickness [11–13]. This is consistent with previous research showing that cortical thickness and surface are two phenotypes highly independent at the genetic level [14], which suggests their alterations could be associated to relatively distinct neuropsychiatric outcomes.

The direction of the association is the same in these studies: low BW individuals show reduced cortical SA. Nevertheless, reductions of cortical SA have been found in different regions when directly analysing the relationship between BW and SA [11, 13], from those when the focus is exclusively on environmental influences on BW, as measured by monozygotic (MZ) twin difference designs [12]. Given that MZ twins have the same genetic background, their phenotypic dissimilarities are believed to be environmentally-induced. Accordingly, the slight contrast in previous reports may indicate that the variety of genetic and environmental factors influencing BW [15,16] can alter cortical anatomy in specific ways. Remarkably, cortical brain surface alterations found by these authors are located in areas with relevance for psychiatric research, such as the temporal, superior frontal and cingulate regions [17–19].

As concluded by Walhovd et al. [13], BW differences across diagnostic groups and conditions may influence differences observed in cortical parameters assessed later in life in neuroimaging research. The present study aims to address three considerations in this regard.

First, it has been reported that human intelligence, measured by IQ, could correlate with brain volume [20,21]. Moreover, altered cognitive capabilities have been related to low BW [22,23]. Skranes et al. [4] reported that cognitive impairments in very-low-BW young adults may be due to decreases in cortical SA caused by altered foetal growth trajectories. It has similarly been proposed that both depressive and anxious pathologies are associated with lower IQ [24], which means both cortical SA and IQ need to be included when analysing the association between BW and cortical variables. Also, a phenotypic correlation between cognitive abilities and cortical SA has been shown in healthy individuals, with genetic factors accounting for ~86% of the association [25]. Hence, genetically informative designs (i.e., studies of MZ twins) may allow us to determine whether the proposed alteration of BW and SA due to uniquely environmental factors [12] holds independently of (likewise) environmental influences on IQ or internalizing psychopathology.

Secondly, there is some–so far inconclusive–evidence of fetal growth alterations as risk factor for adult internalizing disorders (namely, depression and psychological distress) [26,27]. Several cortical morphological brain alterations have been related to these psychopathological states [28–31]. Current evidence suggests that anxious and depressive disorders exhibit a wide degree of comorbidity, a common etiopathology and diagnostic criterion overlap [32–35]. This is probably reflected as shared brain morphometry alterations [36,37] and may also induce SA changes.

Finally, as different patterns of change in cortical and subcortical structures emerge across successive stages of normal development [38], the age range across which the BW-SA link is valid remains unidentified.

Hence, while the previous associations have consistently been demonstrated in children, adolescents and young adults, further verification in older samples is still necessary. Besides, no previous study has evaluated the potential role of anxious-depressive psychopathology in altering these associations.

Our study aims: i) to test the previously identified associations (BW-SA; and MZ differences in both BW and SA) using a sample of middle-aged adults; and ii) to evaluate whether such associations persist regardless of internalizing (anxious-depressive) disorders and differences in IQ profiles.

## Methods

### Ethics statement

Written informed consent was obtained from all participants after a detailed description of the study aims and design, approved by the institutional ethics committee (Comissió de Bioètica de la Universitat de Barcelona (CBUB); Institutional Review Board registry IRB00003099; Assurance number: FWA00004225; http://www.ub.edu/recerca/comissiobioetica.htm). All procedures were in accordance with the Declaration of Helsinki.

### Sample description

Participants of this study were part of a larger twin sample consisting of 242 European descent Spanish adult twins from the general population who gave permission to be contacted for research purposes. The current sample consisted of a 54-individual (27-twin-pair) subset of participants extracted from the initial group. For the current sample, the exclusion criteria applied included age under 18 and over 65, a medical history of neurological disturbance, presence of sensory or motor alterations and current substance misuse or dependence.

Medical records and a battery of psychological and neurocognitive tests were obtained in face-to-face interviews by trained psychologists. Additionally, peripheral blood or saliva samples were obtained from all participants, and zygosity of the pairs was determined by genotyping 16 highly polymorphic microsatellite loci from DNA samples (SSRs; PowerPlex 16 System Promega Corporation). Identity on all the markers can be used to assign monozygosity (i.e., whether twins of a given pair were born from a single fertilized ovum, and are so identical at the DNA sequence level) with greater than 99% accuracy [39].

From the previous sample, a group of 54 middle-aged participants (27 MZ twin pairs; age range 22–56, median age 38; 47% female), who were informative for psychopathology, neurocognition and early stress factors, accepted to participate in an ongoing research project relating cognitive performance, brain function and epigenetic signatures.

The twins included in this subset of 54 participants met the following criteria: a) age at scan between 20 and 56 years, b) both twins right-handed and c) neither twin had a lifetime diagnosis other than depression and/or anxiety. Pairs where one or both twins met the criteria for a lifetime psychiatric diagnosis other than depression or anxiety, or with either neurological or major medical illnesses, were excluded (see *c. Clinical and Neurocognitive Assessment*).

After this point, due to image artifacts and a lack of data on some participants, the final sample (i.e., the subset included in all the statistical analysis) consisted of 48 individuals (24 twin pairs) (mean (SD) age = 36 (11) years; 42% male); there were 6 diagnosis-concordant (anxiety/depression) and 8 diagnosis-discordant pairs, and 10 healthy control twin pairs. All analyses described below refer to this 48-individual sample. Further demographic and descriptive details of this group of twins can be found elsewhere [40] and below.

### Clinical and Neurocognitive Assessment

A trained clinical psychologist applied the Structural Clinical Interview for DSM-IV Axis I Disorders (SCID-I) [41] in a face-to-face interview to screen for the presence of any lifetime depression (major depressive disorder or depressive disorder not otherwise specified) or anxiety spectrum disorders (panic disorder with/without agoraphobia, specific/social phobia, generalized anxiety disorder, agoraphobia without history of panic disorder, anxiety disorder not otherwise specified or obsessive-compulsive disorder).

Individuals meeting the diagnostic criteria for at least one lifetime diagnosis of anxiety or depression were classified as affected by a stress-related disorder, and “concordant”, “discordant” and “healthy” statuses of twin pairs were defined accordingly (Table 1). Most of the affected individuals in this sample experienced a first episode of any anxiety or depressive psychopathology during their adolescence, consistent with previous epidemiological data [42].

**Table 1:** Demographic, clinical, neurocognitive, obstetric and cortical variables for concordant, discordant and healthy MZ twin pairs.

	CONCORDANT (12 subjects)		DISCORDANT (16 subjects)		HEALTHY (20 subjects)		Group comparison
	**Number of individuals**	**%**	**Number of individuals**	**%**	**Number of individuals**	**%**	**X-square** ^***a***^ **; *p***
**Gender (m/f)**	2/10	16.6/83.3	6/10	37.5/62.5	12/8	60/40	5.97; 0.052
**Depression** ^**+**^	4	16.6	4	12.5	-	-	-
**Anxiety** ^**+**^	6	25	1	3.1	-	-	-
**Comorbid** ^**+**^	2	8.3	3	9.4	-	-	-
	**Mean (SD)**	**Range**	**Mean (SD)**	**Range**	**Mean (SD)**	**Range**	**X-square** ^***b***^ **; *p***
**Age**	40.8 (13.3)	23–56	33.1 (12.2)	20–53	35.2 (7.9)	22–48	3.34; 0.188
**IQ**	103 (13.7)	83–127	106.3 (11.6)	87–131	107.4 (6.9)	96–118	0.9; 0.639
**BW (grams)**	2625 (508)	1900–3360	2421 (424)	1800–3000	2482 (536)	1400–3350	1.17; 0.557
**ICV (cm** ^**3**^ **)**	1282 (257)	890–1592	1420 (212)	1106–1829	1533 (78)	1440–1719	9.42; 0.009*
**Total SA (mm** ^**2**^ **)**	152600 (15287)	136200–174500	159900 (17158)	133500–185600	164400 (8658)	151900–182400	3.53; 0.172
**Intrapair BW diff. (grams)**	303 (361)	50–1000	334 (336)	100–1000	315 (277)	0–1000	0.41; 0.82
**Intrapair IQ diff.**	6.7 (7.2)	1–18	6.6 (4.6)	1–13	4.4 (3.9)	0–12	1.16; 0.56
**Intrapair total SA diff. (mm** ^**2**^ **)**	2425 (1960)	144–5399	5874 (5765)	275–17330	3191 (3515)	109–9772	1.22; 0.542

*Abbreviations*: m = males; f = females;

^+^ = lifetime diagnoses according to the Diagnostic and Statistical Manual of Mental Disorders; SD = standard deviation; IQ = intellectual quotient; BW = birth weight; ICV = total intracranial volume; SA = surface area

^*a*^ = X-square and *p*-value estimates for gender data were obtained using Monte Carlo tests with 10^6^ replicates

^*b*^ = Kruskal-Wallis X-square, as these variables were continuous

* = statistically significant *p*-value.

Intelligence quotient (IQ) was estimated from five subtests (block design, digit span, matrix reasoning, information and vocabulary) of the Wechsler Adult Intelligence Scale (WAIS-III) [43,44] by trained psychologists.

Participants were asked to report if they had received pharmacological or psychological treatment or had consulted a psychiatrist or psychologist since they first participated in the study. Only three individuals had life-time exposure to drug treatment for anxiety or depression.

### Birth weight

Information on obstetric complications was collected by direct interviews with the participants’ mothers [45] by means of the Lewis-Murray Obstetric Complications Scale [46]. BW distribution by gestational age of all the subjects in the sample was in accordance with a previous report of Caucasian twins [47].

### MRI acquisition and postprocessing

High-resolution 3D structural datasets, using a T1-weighted magnetization-prepared rapid gradient echo, were acquired at the MRI Unit of the Image Platform (IDIBAPS, Hospital Clínic de Barcelona) by means of a TIM TRIO 3 T scanner (Siemens, Erlangen, Germany), with the parameters: 3D T1-weighted MPRAGE sequence, TR = 2300 ms, TE = 3.03 ms, TI = 900 ms, flip angle = 9°, 192 slices in the sagittal plane, matrix size = 256×256, 1 mm^3^ isometric voxel, 8-channel coil.

MRI scans were processed and analysed using the freely available software FreeSurfer (version 5.1.0; http://surfer.nmr.mgh.harvard.edu/), run on Ubuntu with the Linux 2.6.28-11-generic kernel. Further technical details of FreeSurfer can be found in the literature [48–52].

Cortical SA was measured over the interface between grey and white matter, at the so-called *white matter surface*, as this matches a morphological trait and has lower sensitivity to cortical thickness than the outermost surface [53]. The cerebral cortex was parcellated into 148 units (hereafter regions; 74 per hemisphere) based on gyral and sulcal structure [54]. Cortical SA measurements were obtained for all the subjects for each region and for the total cortical mantle. Regions of interest (ROIs) were defined from previous reports (see *Statistical analysis*), by combining some of the 148 available regions. Afterwards, total intracranial volume (ICV) was estimated [55]. As volume estimates do not increase linearly with SA parameters, ICV was raised to the power of 0.754 (hereafter ICV^0.754^) for later use as a covariate in statistical analysis. Both the cortical parcellation and intracranial volume calculation procedures have been validated against manual measurements (for details, see references above).

These procedures were fully automated; all scans were visually inspected, and slight manual corrections were applied when necessary, following standard procedures. General information on total SA and ICV measurements for the sample are given in Table 1.

### Selection of MRI variables and statistical analysis

Cortical SA across the 148 regions of all 48 subjects were exported as a data matrix, and all statistical analysis was performed in the R Statistical Software [56]. Four different analyses based on multivariate linear regressions were performed. ICV was used as a covariate, given its potential to correlate with general brain features [14].

First, in order to test for associations between raw BW measures and SA phenotypes across the 48 individuals (i.e., considering each twin as an independent observation), linear mixed-effects (LME) models were implemented [57] using SA measurements (in square millimetres) from the dataset mentioned above. LME models allow corrections to be made for the correlated nature of data from twin pairs, thus providing appropriate regression estimates for specific outcomes of interest (here, SA of either total cerebral cortex or each of nine ROIs). Following previous reports on statistical analysis of twin data [58–60], pair id was included as a random effect, to apply a “random” shift in the intercept to both twins in every pair.

Consequently, the initial analysis implemented an LME regression to test for an association between BW, diagnostic status and a measure of total SA, controlling for gender, age, ICV and weeks of gestation [Total SA = *β*_*0*_ + *β*_*1*_(gender) + *β*_*2*_(age) + *β*_*3*_(ICV^0.754^) + *β*_*4*_(weeks of gestation) + *β*_*5*_(BW) + *β*_*6*_(IQ) + *β*_*7*_(diagnosis)].

**Fig 1:**
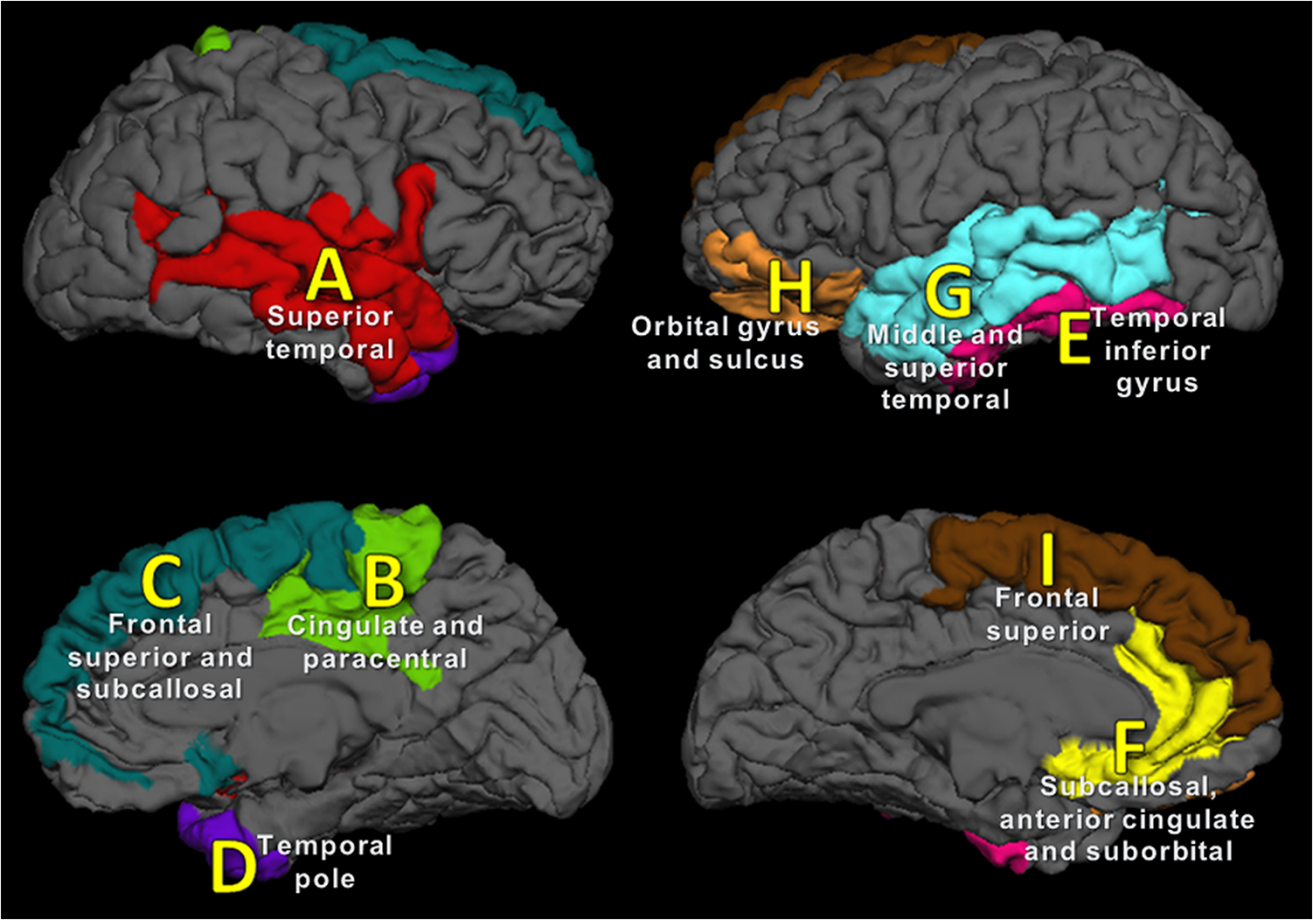
Nine anatomical ROIs for analysis of associations between SA and any of BW, IQ or depression/anxiety. Additional details on ROI selection and nomenclature can be found in *Methods*.

Next, nine similar analyses were carried out using ROIs over relevant Brodmann’s areas [SA of ROI = *β*_*0*_ + *β*_*1*_(gender) + *β*_*2*_(age) + *β*_*3*_(ICV^0.754^) + *β*_*4*_(weeks of gestation) + *β*_*5*_(BW) + *β*_*6*_(IQ) + *β*_*7*_(diagnosis)]. As shown in Fig 1, the ROIs covered, in the right hemisphere, *A*) middle, superior and transverse temporal, inferior insula, orbital medial olfactory and intermediate regions between them; *B*) middle posterior, posterior dorsal and marginalis cingulate regions, plus the paracentral area; *C*) subcallosal and frontal superior gyri, including the suborbital sulcus; and *D*) temporal pole. In the left hemisphere, ROIs were at *E*) temporal inferior gyrus, *F*) a cluster including the subcallosal, anterior cingulate and suborbital regions, *G*) middle and superior temporal cortex, *H*) orbital gyrus and H-shaped orbital sulcus, and *I*) frontal superior region. As discussed below, the Bonferroni correction to the statistical significance threshold was used for this set of regressions (*p*_*Bonferroni*_ = 0.05 / 9 = 0.0056). ROIs *A*, *B*, *C*, *E* and *F* were defined from a study of normal BW variation in the general population [13], whereas ROIs *D*, *G*, *H* and *I* were previously associated with (environmentally-driven) MZ twin pair differences in BW [12]. As discussed above (see *Introduction*), all these candidate areas have been shown relevance for neuropsychiatric outcomes [17–19].

Then, with the aim of evaluating whether lower BW within a twin pair was associated with smaller intrapair SA, regression models using twin pair differences [58,61] were applied. While structural equation modeling allows parsing out the specific genetic, shared and unique environmental factors underlying phenotypic relationships in relatively large samples of both MZ and dizygotic twins [62,63], it is not suited for the current sample with a moderate number of MZ twin pairs. In contrast, other statistical approaches based on regression modeling have been developed to be used with MZ twin data, and their usefulness and feasibility have been proven even for relatively small samples [58,61]. Since previous reports indicate that the association between BW and SA may largely be due to unique environmental factors [12,13], a common method to analyze (environmentally induced) differences within MZ twin pairs was adopted. Briefly, this approach consists in estimating the expected value of an outcome variable from: *E*(*D*_*i*_^*Y*^) = β_W1_ *D*_*i*_^*X*^ + β_W2_ *D*_*i*_^*X’*^ + β_W3_ *D*_*i*_^*X”*^ +…, where the outcome variable (here, intrapair differences in cortical SA) is *D*_*i*_^*Y*^ = *Y*_*i*1_—*Y*_*i*2_; and the individual predictors are in the form *D*_*i*_^*X*^ = *X*_*i*1_—*X*_*i*2_, *D*_*i*_^*X*^ = *X’*_*i*1_—*X’*_*i*2_, and so on, with the set {*X*,*X’*,*X”*,…} being the model predictors (here, ICV, IQ, BW and diagnostic, as mentioned below). The first subindex *i* stands for pair number, with *i* ϵ {1,…,*n*} (here, *n* = 24 MZ pairs), and the second subindex *j* ϵ {1,2} is the randomly assigned co-twin number.

In the previous model, the β_W_ coefficients for covariates with the same value for both co-twins (i.e., age) cancel out by the subtraction *D*_*i*_^*X*^ = *X*_*i*1_—*X*_*i*2_ = 0, and only the variables that may show intrapair differences are thus included.

Initially, total (intrapair) differences in BW and total (intrapair) differences in SA were analysed. In contrast to the previous regressions, neither gender nor age was included as covariates from this point on, as they had the same value for both twins in each pair. A regressor variable corresponding to differences in diagnostic status was included. The models applied to assess intrapair differences included the variables of interest (BW, IQ and diagnostic status) and ICV as a covariate [Intrapair differences in total SA = *β*_*0*_(intrapair differences in ICV^0.754^) + *β*_*1*_(intrapair differences in IQ) + *β*_*2*_(intrapair differences in BW) + *β*_*3*_(intrapair differences in diagnostic)].

Finally, to explore putative locations of origin for this last association, the nine ROIs were evaluated. Intrapair differences were tested following the technique mentioned in the preceding paragraph [Intrapair differences in SA of ROI = *β*_*0*_(intrapair differences in ICV^0.754^) + *β*_*1*_(intrapair differences in IQ) + *β*_*2*_(intrapair differences in BW) + *β*_*3*_(intrapair differences in diagnosis)]. Correspondingly, Bonferroni adjustments were applied by considering *p*_*Bonferroni*_ = 0.05 / 9 = 0.0056.

As only 24 observations were included in the preceding tests of intrapair differences (one observation for each twin pair), *p*-values for this regression model were obtained from permutation tests, with the *lmPerm* R package [64]. Such permutation-based *p*-values are particularly suited to saturated experimental designs and datasets from non-normal populations or those with apparent outliers. Permutated *p*-values shown in the *Results* were in agreement with those obtained with ordinary least squares regressions.

## Results

Table 1 shows descriptive sample data, arranged according to the psychopathological status of each twin pair (*concordant*, *discordant* or *healthy* pairs).

Among the three groups (*concordant*, *discordant* and *healthy*), no differences were found for either age, BW, IQ or total SA. ICV did show statistically significant inter-group differences (*p* = 0.009), which were seemingly driven by the *concordant* group (Kruskal-Wallis X-square for *discordant* vs. *healthy p* = 0.286), whose lower ICV mean value might have been due to the fact that there were 5 female and only 1 male pair. Consequently, adjustments for ICV were included in all subsequent tests (see *Method*s).

As a preliminary step, collinearity between BW and IQ, BW and diagnosis, and IQ and diagnosis were tested; none of them was found to be statistically significant.

### BW and cortical SA: direct association

When evaluating all 48 observations independently (i.e., correcting for the clustered origin of observations from twin pairs), we found associations between total cortical SA and both BW (*β* = 5.89, *t* = 2.79, *p* = 0.011) and IQ (*β* = 299.2, *t* = 2.67, *p* = 0.015). Nonetheless, total SA was not related to diagnosis of internalizing psychopathology (*β* = -2279.9, *t* = -1.52, *p* = 0.145).

By examining the nine ROIs described above, it was found that the size of only one of them was associated with BW. Specifically, the dimension of ROI *B*, in the right cingulate and adjacent areas positively correlated with BW (*β* = 0.34, *t* = 3.39, *p*_*Bonferroni*_ = 0.026). Similarly, IQ score was directly proportional to size of the left subcallosal, anterior cingulate and suborbital cluster (ROI *F*, *β* = 14.1, *t* = 3.27, *p*_*Bonferroni*_ = 0.034) and also showed a trend towards association with the left temporal inferior gyrus (ROI *E*, *β* = 13.5, *t* = 2.79, *p*_*Bonferroni*_ = 0.099) (see Fig 1). No association was found between the size of any of these ROIs and diagnostic status.

### Intrapair differences in BW and intrapair differences in cortical SA

Within a twin pair, smaller total cortical SA was associated with lower BW (*β* = 7.6, *p* = 0.004), but with no differences in either IQ (*β* = 125.5, *p* = 0.2) or diagnostic status (*β* = -1677.3, *p* = 0.149).

Finally, analysis of the nine ROIs detailed above showed that smaller intrapair area in the left middle and superior temporal cortical regions was related to lower BW within a pair (ROI *G*, *β* = 0.8, *p*_*Bonferroni*_ = 0.029) (see Fig 1). Intrapair differences in IQ and diagnosis had no effect on intrapair SA differences in these ROIs.

## Discussion

These results, from a middle-aged adult sample, support an association between low BW and reduced cortical SA, in line with previous findings in younger samples. Such an association was found at the level of both individuals and MZ-differences, and by evaluating both total and ROI SA. Neither the internalizing (i.e., anxious-depressive) psychopathological status nor the IQ scores of the participants altered this association.

### BW and cortical SA

Initially, analysis was performed in order to search for influences of BW, IQ and diagnosis on cortical SA, across the 48 participants. BW was related to SA of the whole cortical mantle–each gram of BW accounted for approximately 5.94 mm^2^ of adult SA–and of a region comprising the right cingulate and paracentral cortex. IQ was also associated with SA, in agreement with former publications showing larger brain volumes in people with higher IQ [20,21]. Specifically, higher IQ was correlated with larger total SA and larger SA of two regions in the left hemisphere: temporal inferior cortex and a cluster including cingulate, subcallosal and suborbital areas.

The analysis considered all subjects independently (i.e., as members of a general-population sample, correcting for the clustering of observations due to twin-pair relatedness). Remarkably, all associated ROIs (*B*, *E* and *F*, see Fig 1) corresponded to those derived from a study of a heterogeneous sample of healthy non-related individuals [13].

### Intrapair differences in BW and intrapair differences in cortical SA

As mentioned above, it is known that BW and SA are the result of both genetic and environmental influences. Accordingly, by examining MZ twin pair differences, additional analysis explored the relationship of exclusively environmental effects on both phenotypes. We found that, within a twin pair, lower BW is associated with smaller total SA. Every gram of intrapair disadvantage in BW was associated with an average reduction of approximately 7.6 mm^2^ in total cortical SA in adulthood. By comparing this result with those of previous sections, it is feasible to infer that SA is particularly sensitive to environmentally-driven BW variation. ROI analysis of intrapair differences indicated that a region covering the left middle and superior temporal cortex was specifically susceptible to environmental factors affecting BW. This area (ROI *G*, see Fig 1) was defined from a previous study of small intrapair BW differences of MZ twins [12].

While it was possible to show that non-genetic influences on foetal growth provoke changes in brain morphology, this was not the case for IQ. The detected IQ-SA link may be due to either familial factors (genes and shared environment) or gene-environment interactions, but solely environmental effects on both IQ and SA (as detected by MZ pair differences) were not associated with each other. Notably, this is also consistent with previous indications of the genetic origins of the association between intelligence and brain volume [21] and more recent evidence that most of the cognitive ability-SA relationship may be accounted for by genetic factors [25]. Our results are in agreement with these studies and also suggest the existence of environmental factors that commonly affect BW and SA, but not IQ and SA.

Lastly, all these relationships persisted independently of diagnosis of anxious-depressive disorders; this implies the results are robust, despite a putative confounding effect of clinical traits. Although inconclusive, there is some evidence linking foetal growth and risk for adult depression and/or psychological distress [26,27]. Hence, one could expect some differential brain morphological effect depending on diagnostic criteria. Nevertheless, this was not the case in the analysis carried out here: our results indicate that BW alters SA regardless of internalizing psychopathology traits. Further research using distinct severity of psychopathological status may clarify potential diagnosis-specific effects.

### Limitations of the study

Finally, some limitations deserve consideration. First, the sample size is relatively small. Though replication using larger independent samples and with more severe phenotypic discordance is required, it is worth noting that the current findings are consistent with previous reports that show a long-lasting influence of early foetal growth alterations on adult brain morphology, which persist even despite the presence of psychotic psychopathology [11–13]. The present results partly replicate such studies in an independent sample, and suggest that the BW-SA relationship holds despite the presence of anxious/depressive disorders. This agreement probably suggests the presence of strong effect sizes for the above mentioned relationships.

Other putative limitation of this work is the phenotypical (i.e., clinical) heterogeneity across MZ twin groups, with an unbalanced distribution of concordant, discordant and healthy pairs. While cross-validating the present results with larger independent datasets from twin pairs with a narrower and more balanced phenotypic distribution is necessary, two features from the ongoing study should be noted. First, the clinical phenotype did not seem to modify any of the statistical associations described here. Namely, both BW-SA and IQ-SA associations were statistically significant across the diverse clinical-psychopathological composition of the MZ subgroups: the associations held across the set of all concordant, discordant and healthy pairs.

In addition, the only IQ measure employed here was derived from a full-scale assessment. While previous research indicates that both full-scale and performance IQ may be related to differences in BW of MZ co-twins [12], exploring the associations between performance IQ and BW may be difficult here mainly due to two reasons. First, using only a few intelligence subscales to build up a performance IQ measure may give rise to statistical distributions departing from normality. In this sense, the full-scale IQ measure was computed by averaging over a relatively large number of intelligence subscales, thus approaching a robust and normally-distributed variable. Secondly, in the larger UB twin registry dataset (*n* > 200 co-twins), not all IQ subscales seem associated with BW, seemingly due to the moderate (average) intrapair difference in BW. Importantly, the associations described here between cortical SA and IQ are in agreement with former reports and show consistency with biological mechanisms proposed by recent literature.

## Conclusion

The present study supports previous findings indicating that BW has a long-lasting effect on cortical SA, where a mixture of familial (genes and shared environment) and solely environmental interactions may influence both foetal growth and brain morphology; and environmental factors affecting BW have a specific effect on SA as well: a portion of SA which is entirely driven by the environment seems to be modified by the fraction of BW that is also determined by non-genetic influences. This distinction is particularly interesting given that ROI analysis indicate that the left temporal cortex is sensitive to environmental influences on BW, but it is not determined by the whole BW variation; which indicates that diverse determinants of BW (genes, environment and their interplay) may affect SA differently. Additionally, higher IQ scores correlate with larger SA; this relationship does not seem to be driven by unique environmental factors. None of these associations were modified by the presence of internalizing (anxious-depressive) disorders.
